# LDH-A Acetylation: Implication in Cancer

**DOI:** 10.18632/oncotarget.1007

**Published:** 2013-04-28

**Authors:** Di Zhao, Yue Xiong, Qun-Ying Lei, Kun-Liang Guan

**Affiliations:** Molecular and Cell Biology Laboratory, Institutes of Biomedical Sciences, Fudan University, Shanghai, China; Department of Biochemistry and Biophysics, Lineberger Comprehensive Cancer Center, University of North Carolina at Chapel Hill, NC; Molecular and Cell Biology Laboratory, Institutes of Biomedical Sciences, Fudan University, Shanghai, China; Department of Pharmacology and Moores Cancer Center, University of California San Diego, La Jolla, CA

Upregulation of lactate dehydrogenase A (LDH-A) is commonly observed in many tumor types. Previous studies have revealed LDH-A transcriptional activation by the increased activity of Myc and HIF in human cancers. Is LDH-A regulated by post-translational modifications during tumorigenesis? If so, can such knowledge be used to assist cancer early diagnosis and treatment?

**Figure 1:**
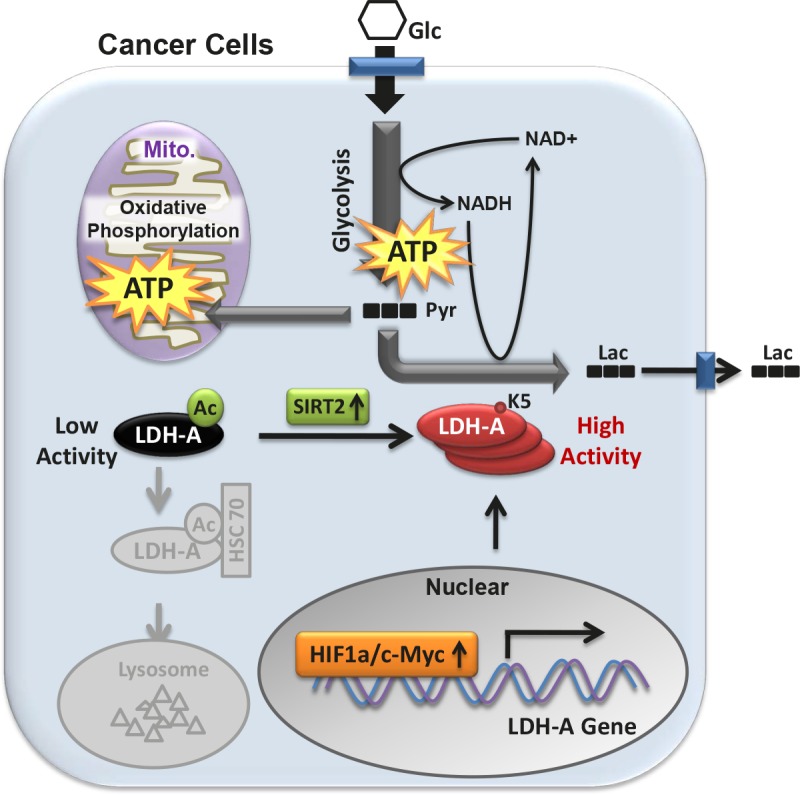
Acetylation at K5 inhibits LDH-A enzyme activity and promotes its lysosomal degradation SIRT2 deacetylates LDH-A and increases its activity and protein level. The transcription of LDH-A is stimulated by Myc and HIF in some cancer types. Glc, glucose; Pyr, pyruvate; Lac, lactate; Ac, acetylation; Mito., mitochondria.

Reprogramming of energy metabolism, particularly the elevated glucose uptake, glycolysis and lactate production, is a hallmark of cancer. In order to support rapid cancer cell growth, glycolysis is highly elevated to provide metabolic intermediates for macromolecule biosynthesis. Instead of entering mitochondria to fuel the tricarbolic acid (TCA) cycle and oxidative phosphorylation for efficient energy production, a large fraction of pyruvate in cancer cells is converted into lactate by LDH, accompanied by NAD+ regeneration to maintain high glycolysis rate (Figure1). Moreover, the excess lactate transported out of cytoplasm may condition the microenvironment, which promotes interaction between cancer cells and stromal cells, eventually resulting in increased cancer cell migration and invasion.

LDH is a homo- or hetero-tetrameric enzyme consisting of two different subunits encoded by the highly related genes, LDH-A and LDH-B. Both LDH-A and -B catalyze the reversible conversion between pyruvate and lactate using NAD+ as a cofactor. However, LDH-A favors the conversion of pyruvate into lactate, while LDH-B prefers the inverse reaction. Actually, it has long been known that many tumor cells express high levels of LDH-A, including non-small-cell lung cancer, colorectal cancer, and breast cancer. In many tumors, elevated LDH-A levels have been correlated with poor prognosis and resistance to chemotherapy and radiotherapy. It has been reported that inhibition of LDH-A by either RNA interference or pharmacological agents blocks tumor progression in vivo, supporting an important role of elevated LDH-A in tumorigenesis and LDH-A as a potential therapeutic target.

Due to the critical function of LDH-A in tumor metabolism, researchers are eager to know how LDH-A is regulated in cancer cells. It has been reported that elevated activities of c-Myc or HIF1a transcription factor contribute to the increased LDH-A expression in some cancer types. Recently, our group has demonstrated a mechanism of LDH-A up-regulation by post-translational modification in pancreatic cancers (Zhao, et al., Cancer Cell, 23, 464-476, 2013). We found that LDH-A is acetylated at lysine 5 (K5) and this acetylation reduces LDH-A catalytic activity. Furthermore, acetylation decreases LDH-A protein level. The K5-acetylated LDH-A is recognized by the HSC70 chaperone and delivered to lysosomes for degradation (Figure 1). Replacement of endogenous LDH-A with an acetylation mimetic mutant decreases cancer cell proliferation and migration, indicating a critical role of LDH-A acetylation in cell growth control. Importantly, K5 acetylation of LDH-A is reduced and accompanied with increased LDH-A protein levels in both early and late stages of pancreatic cancers. Our data suggest a possible role of K5 acetylation contributing to pancreatic cancer initiation, but not progression.

Pancreatic cancer, the eighth most common cause of cancer-related death worldwide, has an extremely poor prognosis: for all stages combined, the 1- and 5-year survival rates are 25% and 6%, respectively; while the median survival for metastatic disease is about 6 months. For most pancreatic cancer patients, they are usually diagnosed at late stages with metastasis and have limited options for treatment. The effect of chemotherapy/radiotherapy on pancreatic cancer is rather poor. Thus, early diagnose is critical for pancreatic cancer patients to have a time window for treatment. The current diagnosis depends on the descriptions of symptoms, computed tomography (CT scan), magnetic resonance imaging (MRI), ultrasound, and positron emission tomography (PET scan). A definite diagnosis is by biopsy, such as percutaneous needle biopsy. Therefore, more convenient and credible early diagnosis is urgently needed for pancreatic cancer.

Because elevated LDH-A is detected in almost every type of cancer, it is one of the first tumor markers to be introduced into clinical practice. LDH-A has been used to monitor treatment of some cancers since its correlation with poor prognosis and chemotherapy/radiotherapy resistance. Although we found LDH-A K5 acetylation is reduced in pancreatic cancer, we failed to detect a correlation between decreased K5 acetylation and liver cancer initiation. These observations indicate that K5 acetylation of LDH-A could be a marker for some cancers, such as pancreatic cancer, but not others, such as liver cancer. Given the fact that LDH-A K5 acetylation can be readily detected by specific antibody, it may serve as a valuable marker for diagnosis of some cancers. We further speculate that LDH-A K5 acetylation labeling coupled with PET/CT would be a potential early diagnose marker for pancreatic cancer. Further investigation to solidify the correlation between K5 acetylation of LDH-A and tumor initiation of various cancer types is needed before LDH-A K5 acetylation could be considered as a general cancer marker.

LDH-A has been considered as a potential therapeutic target, based on its vital function in sustaining high rate of glycolysis in cancer cells. Indeed, LDH-A inhibitors and siRNA inhibited tumor growth in mouse models. Considering the inhibitory effect of K5 acetylation LDH-A, drugs that stimulate LDH-A acetylation by targeting the LDH-A acetyl transferase or deacetylase should inhibit LDH-A, therefore may have therapeutic value for cancers with high LDH-A activity. Taken together, our recent study not only demonstrates a novel mechanism of LDH-A regulation, but also provides a potential early diagnosis maker and therapeutic target for pancreatic cancer.

